# Complex AC Magnetic Susceptibility as a Tool for Exploring Nonlinear Magnetic Phenomena and Pinning Properties in Superconductors

**DOI:** 10.3390/ma16144896

**Published:** 2023-07-08

**Authors:** Krastyo Buchkov, Armando Galluzzi, Elena Nazarova, Massimiliano Polichetti

**Affiliations:** 1 Institute of Solid State Physics, Bulgarian Academy of Sciences, 72 Tzarigradsko Chaussee Blvd., 1784 Sofia, Bulgaria; nazarova@issp.bas.bg; 2 Department of Physics ‘E.R. Caianiello’, University of Salerno, Via Giovanni Paolo II, 132, I-84084 Fisciano, Italy; mpolichetti@unisa.it; 3 CNR-SPIN Salerno, Via Giovanni Paolo II, 132, I-84084 Fisciano, Italy

**Keywords:** AC magnetic susceptibility, pinning energy, second harmonic, third harmonic

## Abstract

The versatile AC magnetic susceptibility technique offers a detailed insight into the complex electrodynamic phenomena in superconductors. In the present study, we outline the key effects related to the temperature, AC field amplitude and frequency variations of the fundamental and harmonic components for an investigation of the vortex dynamics in a flux-grown FeSe crystal. By means of higher harmonic (nonlinear) analysis, we have explored certain atypical, asymmetric features in the AC magnetic response. These effects were identified through the detection of an even (second) harmonic and an unusual temperature shift in the odd (third) harmonic, possibly due to the complex interactions related to the composite superconducting/magnetic morphology of the crystal. Using the high-frequency sensitivity of the third harmonic, the basic functional dependencies of the pinning activation energy, as the main mixed state parameter, were determined with the implementation of the Kim–Anderson Arrhenius relation in the framework of the collective creep theory.

## 1. Introduction

AC magnetic susceptibility (ACMS) is a versatile magnetometric method for a comprehensive analysis of all electrodynamic (Meissner effect, intermediate and mixed state) properties of superconducting materials, in particular, the critical parameters, pinning, dissipation processes, diamagnetic shielding fraction and domain (inter- and intragranular) morphology, and for the detailed analysis of AC losses in superconducting single-crystals, polycrystals, bulks, films and tapes [1,2,3,4,5,6]. 

The physical phenomenology of the technique is built upon the phase-sensitive detection of the complex fundamental and higher harmonic ACMS components, which are directly related to the magnetic energy absorbed (in-phase) or dispersed as AC losses (out-phase) by the sample during the AC field cycle. This allows high measurement precision (accuracy and resolution) close to the performance of AC/DC SQUID magnetometers. Another advantage is the high sensitivity of the ACMS harmonic components to the broad set of experimental degrees of freedom such as the temperature (T), the amplitude ($H_{ac}$) and frequency (f) of the AC magnetic field, the bias DC field (${\mu_{0}H}_{dc}$) and the sample geometry [7,8,9,10,11]. 

The combined analysis of the fundamental and higher (odd and even) harmonic components is one of the advanced ACMS applications for the determination of the main critical parameters of a superconductor. It provides a detailed insight for studying both the linear (reversible) and nonlinear (hysteretic) processes [7,12,13,14,15,16,17,18,19] in the magnetization, particularly the flux pinning in the critical state and the dissipation processes coming from the vortex relaxation by flux creep and, depending on the experiment conditions, also by the thermally assisted flux flow and the flux flow with or without an imposed DC field.

We can extend the list of phenomena accessible by the ACMS considering the Bean-Livingston, edge and geometrical barriers [20,21,22,23], the Josephson effects [24], the vortex avalanches [25] and the Campbell regime [16] (in the presence of the reversible oscillation of the vortices inside the pinning potential wells). 

The increase in the AC field frequency leads to a growth in the induced electric field E of the superconductor and an overall change in the electric field vs. current density $E\left(J\right)$ characteristics [7,14,26], which is correlated with an increase in dissipative process activity, especially in the case of the irreversible thermally activated flux creep. The AC susceptibility experiment allows measurements with both high-resolution criteria and a wide range of variation in E by changing the frequency and amplitude of the applied magnetic field [27,28]. Therefore, it is used as a complementary method for the analysis of the vortex dynamics to other techniques such as resistive transport, static and dynamic magnetic relaxation by means of SQUID and conventional (vibrating sample) DC magnetometry and local methods such as scanning Hall probes [29].

The consequent phase lag in the AC magnetic response results also in shifting effects of the fundamental and higher harmonic components which cannot be explained in the frames of the critical state models [7,30]. Therefore, the ACMS analysis of the total AC loss ${\chi \prime \prime }_{1}$ peak at different frequency ranges is established as a customary routine for the study of the dynamical regimes [31] and especially of the pinning energy $U_{p}$ and the mechanisms defined by the defect structure—2D/3D, single/collective, etc. [32,33,34,35,36]. Therefore, it is widely applied for the analysis of the pinning characteristics of various conventional and nonconventional superconducting systems. 

The experimental parameter variations ($H_{ac}$, f and ${\mu_{0}H}_{dc}$) have a much more prominent effect on higher order harmonic behavior than on the fundamental susceptibility. Correspondingly, they are developed as an auxiliary tool to separate the contributions, especially of the nonlinear dynamical processes. Following this context, by means of ACMS fundamental and harmonic analysis, we have performed an initial mixed state characterization of a flux-grown superconducting FeSe crystal. The composite superconducting/magnetic morphology of the investigated crystal results in a complex ACMS behavior. Several novel atypical features were observed by comparing the temperature-divergence effects in the fundamental and third harmonic (and even second) generation. The temperature and critical current functional dependencies of the pinning energy were determined via a simplistic Kim–Anderson Arrhenius approach rarely applied in the frames of the third harmonic. 

The presented analysis also overviews the phenomenological basis that determines the integral behavior of the fundamental, even (second) and odd (third), harmonic components which form almost the entire AC magnetic response of a given superconducting sample and aims to expand the useful qualitative and quantitative characterization capabilities of the ACMS technique.

## 2. Materials and Methods

The plate-like FeSe crystals (sample dimensions: *a* ≈b=2 mm and d=0.15 mm) were grown using a high-temperature flux method with NaCl/KCl salts as the eutectic agents. The specific details of the growth procedure and the structural and chemical characterization were described elsewhere [37]. This primary compound is a representative of the large group of iron-chalcogenide-based unconventional superconductors [38].

It is important to note that the comprehensive composite morphology of the crystal resulted from the two-phase cogrowth, i.e., superconducting tetragonal (P4/nmm space group) and ferromagnetic hexagonal (P3(1)21 space group). The phases coexist in a complex structural configuration which affects both the superconducting and magnetic nature [39]. 

The ACMS experiments were performed using the AC/DC magnetometer module of the PPMS-9T Quantum Design^@^ cryostat system [40]. The conventional AC magnetic susceptometer design is built upon the principles of the mutual-inductance bridge, which consists of a coaxial coil-set system, with a primary field-excitation coil together with a connected in series opposition secondary pickup signal detection coil and a compensation coil.

The measurement series were conducted in the temperature range 2.5–15 K with an ACMS scan step of 0.1 K. The main experimental series were performed without an imposed high DC field and under zero-field cooling conditions to maintain a diamagnetic state in the starting thermomagnetic history of the sample with an initial 30 min period of thermal stabilization.

In order to increase the measurement accuracy and sensitivity prior to every scan point, the temperature was stabilized to keep a thermal equilibrium of the sample and the magnetometer, together with a 5-point signal-averaging (in the coil-set assembly) and phase-calibration routine. The precise phase adjustment is vital for the extraction of higher harmonics since the eventual phase errors will cause (multiplied by the harmonic number) significant AC susceptibility deviations. The implemented digital signal processing (DSP) allows the simultaneous data acquisition of all odd and even (up to the 9th) harmonic components. For a convenient comparison, we have presented the data in both CGS and SI unit systems. 

We have to point out the importance of the complex susceptibility notation $\chi_{n}=\chi_{n}^{\prime }\pm {i\chi }_{n}^{\prime \prime }$ (see the milestone article of Ishida and Goldfarb, appendix in [3,41]) which could result in a difference in the sign (+/−) or interchanged in-phase and out-phase components for the odd and even harmonics. These considerations are important for direct experimental and theoretical model comparisons. 

The higher harmonics can be indirectly affected by the eventual remanent field trapped in the superconducting PPMS magnet or altogether from the Earth’s magnetic field. The behavior of the even order components is determined by the specific $H_{ac}/{(\pm \mu_{0}H}_{dc})$ ratio $\left(\mu_{0}=1\;(CGS\right)$; $4\pi \times 10^{-7}Wb/A\cdot m\;(SI)$ as the magnetic permeability of the vacuum). Therefore, special care is taken to reduce the influence of the frozen bias field. A conventional “demagnetization” routine was implemented, gradually reversing and decreasing the value of ${\mu_{0}H}_{dc}$ down to ~1 Oe (80 A/m). Furthermore, the cryostat system was equipped with an additional “ultra-low field” coil-set option used for the compensation/cancelation of the residual trapped flux and to effectively minimize the bias field to less than ${\mu_{0}H}_{dc}\;\sim \;0.05\;Oe\;(4\;A/m)$.

The AC magnetic field was applied perpendicular to the crystal plane; however, due to the structural complexity and lattice reorientation, this corresponds to a $H_{ac}⊥[101]$ crystallographic plane [42] of the tetragonal superconducting β-phase. Therefore, the field profile of the sample is also distorted and the significant geometrical effects in the evaluation of the critical parameters have to be considered. 

## 3. Results and Discussion

In order to investigate the influence of the different processes on the magnetic response and to evaluate the characteristic temperature and critical current dependencies of the pinning potential $U_{p}$ for the FeSe crystal, we have performed an extended measurement series of the ACMS components as a function of the temperature with a variation within a broad instrumental range of applied AC magnetic field amplitudes: $H_{ac}$ = 0.5 Oe (40 A/m), 1 Oe (80 A/m), 2 Oe (159 A/m), 4 Oe (318 A/m), 8 Oe (637 A/m), 12 Oe (954 A/m) and frequencies f=10.7, 107, 333, 777, 1077, 3333, 5385, 7777, 8321, 9693 Hz. 

It is both an experimental and analysis challenge to determine the parametric tendencies considering the sensitivity of the second and third harmonics compared to the fundamental component, altogether with a low signal intensity due to the small crystal size at the lowest AC amplitude and frequency range. This is especially valid for the even harmonics, where for purpose of clarity, it was necessary to implement additional data processing with noise filtering by means of a fast Fourier transform (FFT). 

**Figure 1:**
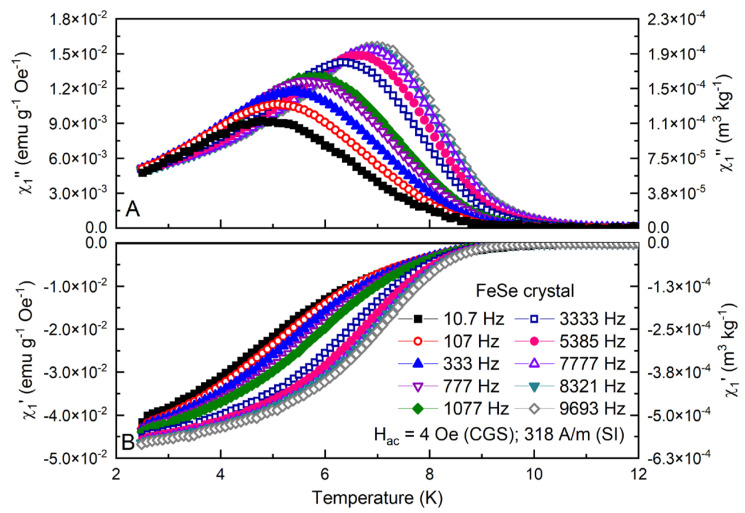
Temperature dependence of the out-phase ${\chi \prime \prime }_{1}$ (**A**) and in-phase ${\chi \prime }_{1}$ (**B**) components at fixed $H_{ac}=4\;Oe\;(318\;A/m)$ and different frequencies for the FeSe crystal.

The typical temperature dependencies of the fundamental components for a fixed applied $H_{ac}=4\;Oe,\;(318\;A/m)$ and for frequencies in the range of [10.7–9693 Hz] are presented in Figure 1A,B. The critical temperature is determined to be $T_{c}\;\sim \;10.5\;K$, marked by the first negative diamagnetic signal detection at $H_{ac}=1\;Oe,\;(80\;A/m)$ and f=777 Hz.

Expectedly, the frequency increase causes a significant temperature shift in the superconducting transition and in the position and a further intensity increase in the AC loss peak ${\chi \prime \prime }_{1}$ under the influence of the dissipation processes (including conventional eddy currents). The vortex dynamic phenomena are especially prominent in the cuprates, in the iron-based chalcogenides and in the pnictides as a typically extreme type-II superconductor [43,44,45,46]. Generally, these are thermally activated and/or Lorentz force triggered to move/displace the vortices through the pinning environment. Even at the lowest temperature interval, due to the flux creep, there is a gradual (time-dependent) flux diffusion into the superconductor and a general reduction in the effective critical current density. 

During the AC field cycle in the typical 10 Hz–10 kHz ACMS working range, flux creep activity varies in the time scale of milli- to microseconds. At higher frequencies, the time window for flux-front penetration is far more limited, resulting in an indirect improvement in the shielding ability and overall shifting of the diamagnetic transition to higher temperatures.

This approach is also suitable to probe the vortex response even at very low currents or to prompt reversible vortex oscillations (inside or between the pinning centers) at ultrahigh frequencies [47]. The fundamental AC loss peak ${\chi \prime \prime }_{1}$ marks the total dissipation (both linear and nonlinear) at the point of full penetration of the applied field. 

**Figure 2:**
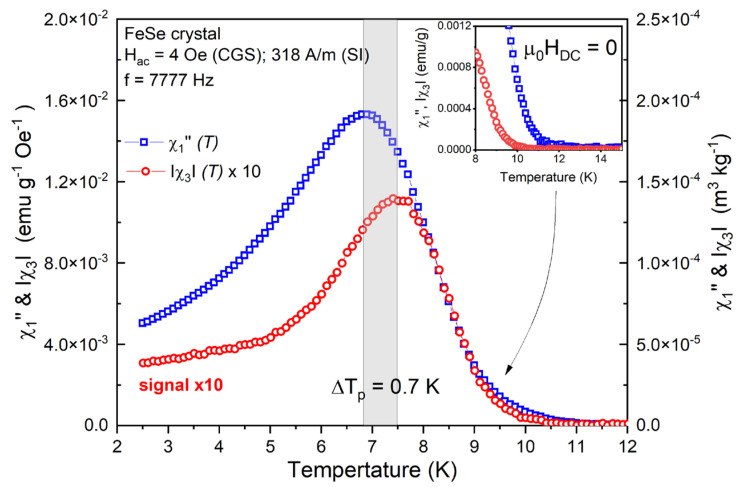
Scaled comparison of the temperature dependence of ${\chi \prime \prime }_{1}$ and $\left|\chi_{3}\right|$ (multiplied by 10 to scale) at fixed $H_{ac}=4\;Oe\;\left(318\;A/m\right)$ and f=7777 Hz for the FeSe crystal. The inset section shows an enlarged view of both the components close to the critical temperature.

However, the higher harmonics are entirely related to the activity of the nonlinear processes [48,49,50]. We present a scale comparative view of the ${\chi \prime \prime }_{1}$ and $\left|\chi_{3}\right|$ maxima in Figure 2 at $H_{ac}=4\;Oe\;(318\;A/m$), f=7777 Hz. The ${\chi^{\prime \prime }}_{1}(T)$ and $\left|\chi_{3}\right|(T)$ peak signal intensity difference is significant—about one order of magnitude, which identifies the dominant contribution of the linear processes. Based on this difference, the combined fundamental and higher harmonic analysis is a powerful approach to examine the broad spectrum of processes that form the AC magnetic response. In the same context, unusually for these experimental conditions ${(\mu }_{0}H_{DC}=0\;Oe)$, a significant separation in the temperatures of the fundamental and third harmonic maximum together with a shift of the $\left|\chi_{3}\right|(T)$ peak to a higher temperature was observed. This marks a certain divergence in the activity of the linear/nonlinear phenomena. In general, such a temperature disparity is determined by the flux-creep contribution over the harmonics in the case when $\mu_{0}H_{DC}\gg H_{ac}$ [13]. An opposite tendency is observed close to $T_{c}$ (shown in the inset section), also marking a narrow temperature interval (${\chi \prime \prime }_{1}>0$ and $\left|\chi_{3}\right|\approx 0$) where the linear dissipative processes are dominant. 

**Figure 3:**
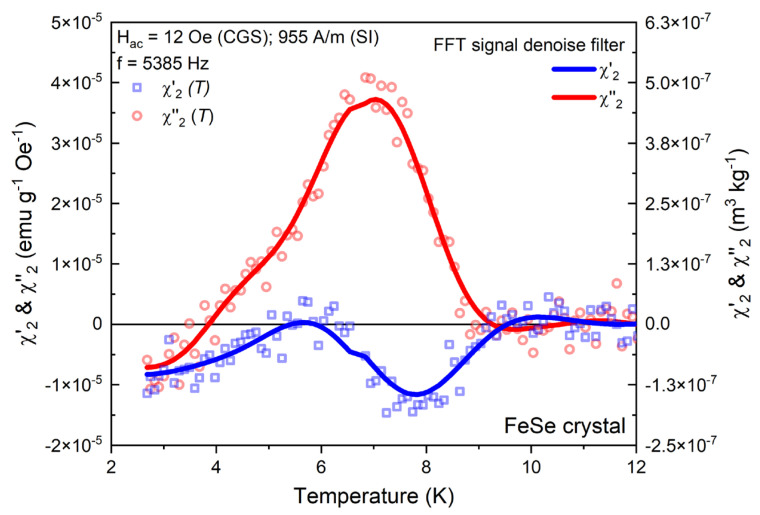
Temperature dependence of the even-harmonic ${\chi \prime }_{2}$ and ${\chi \prime \prime }_{2}$ at fixed $H_{ac}=4\;Oe\;(318\;A/m)$ and f=5385 Hz for the FeSe crystal. For the purpose of clarity, an FFT noise-filtering routine is implemented.

**Figure 4:**
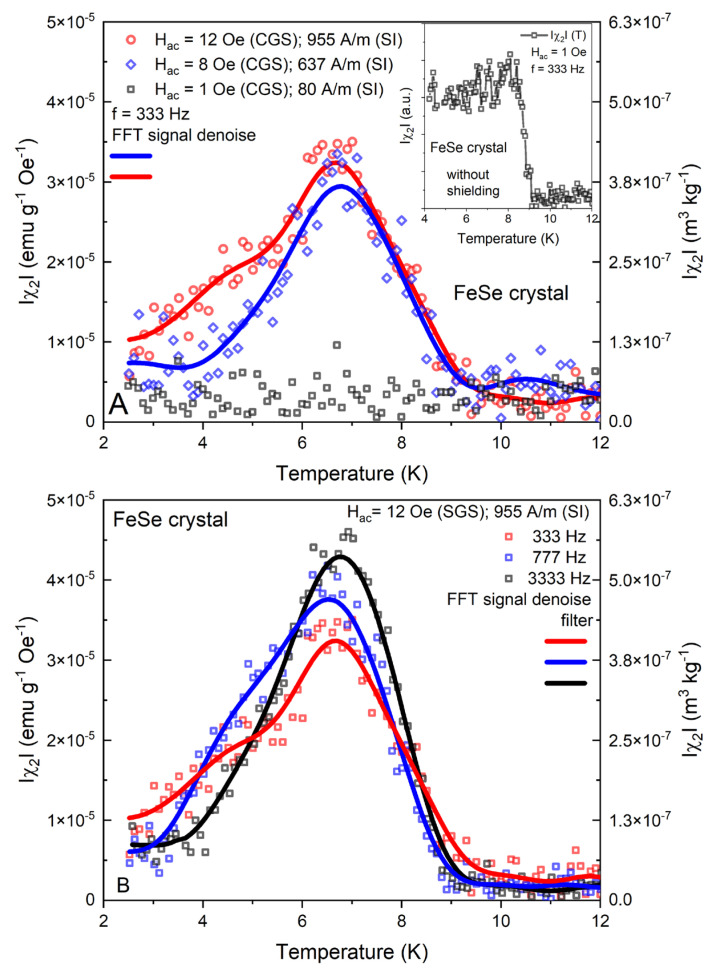
Temperature dependence of $\left|\chi_{2}\right|$ at different amplitudes and fixed frequency f = 333 Hz (**A**) and at various frequencies, f=333, 777 and 3333 Hz, at fixed $H_{ac}=12\;Oe\;\left(955\;A/m\right)$ (**B**) for the FeSe crystal. In the inset is presented the corresponding even-harmonic signal at $H_{ac}=1\;Oe\;(80\;A/m)$, f=333 Hz without trapped field compensation.

**Figure 5:**
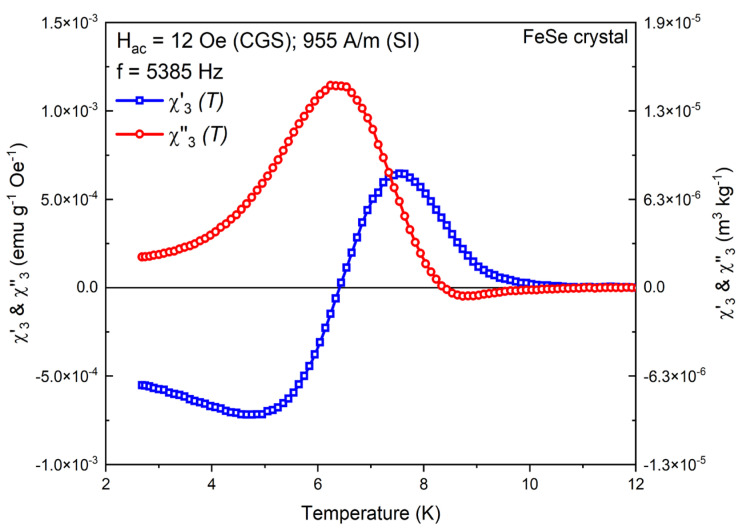
Temperature dependence of the odd-harmonic in-phase ${\chi \prime }_{3}\left(T\right)$ and out-phase ${\chi^{\prime \prime }}_{3}(T)$ components at fixed $H_{ac}=12\;Oe\;(955\;A/m)$ and f=5385 Hz for the FeSe crystal.

Following an analogous context, another intricate feature of the AC magnetic response is revealed by the detection of the second order harmonic (as shown in Figure 3, Figure 4 and Figure 5), which also is an unusual observation taking into account the employed experimental conditions with minimized DC field effects. The detected signal is very close to the sensitivity limit of the instrument and significantly lower compared even to the third harmonic. The even-order harmonic generation is associated to a particular case when there are both superimposed $H_{ac}≅{\mu_{0}H}_{dc}$ amplitudes within a certain equality balance of the magnitudes of the alternating and bias static-field vectors [51,52]. The resulting altered waveform creates an asymmetric AC field cycle (hysteresis loop) which leads to a more complicated critical current field dependency in the superconductor. Therefore, the analytical basics of the even harmonic behavior are mostly developed under the framework of the generalized version of the Bean and Kim–Anderson critical state models [51,52].

Additionally, the second harmonic in the susceptibility model is a prominent characteristic in various granular superconducting systems analyzed in the context of Josephson network models [24,53,54,55]. Nevertheless, the complete phenomenological aspects and underlying processes related to the origin of even harmonic generation are vastly unexplored, with quite few experimental or numerical studies in the literature regarding the ACMS parametric variations. 

The temperature dependence of the in-phase ${\chi^{\prime }}_{2}(T)$ and out-phase ${\chi ”}_{2}(T)$ components for the investigated FeSe crystal at a fixed applied $H_{ac}=12\;Oe\;(954\;A/m)$ and f=5385 Hz is presented on Figure 3. 

Usually, high order harmonics have more complex oscillating behavior (compared to the fundamental), dynamically ranging from positive to negative values depending on the underlying processes in the AC magnetic response. Identical curve shapes are also predicted by the theoretical basis of the Kim–Anderson and generalized Bean critical models [51,52]. 

The influence of the amplitude [$H_{ac}=1\;Oe\;\left(80\;A/m\right),\;8\;Oe\;\left(637\;A/m\right)$ and 12 Oe (954 A/m)] and frequency [f=333, 777, 3333, 5381 Hz] variation over the temperature dependence of the main peak of the $\left|\chi_{2}\right|$ modulus is presented in Figure 4A,B. As we noted in the previous paragraphs, the signal intensity of the second harmonic is a highly dependent (intensify or decrease) function of the $H_{ac}/{\mu_{0}H}_{dc}\to 1$ equality ratio and the resulting waveform asymmetry in the superconductor’s AC magnetization cycle. We can presume that the observed tendency of the $\left|\chi_{2}\right|$ signal reduction down to the noise level with the $H_{ac}$ decreasing from $12\;Oe\;\left(954\;A/m\right)\;to\;1\;Oe\;\left(80\;A/m\right)$ marks an evident restoration of the broken symmetry at the lowest applied amplitude (as seen in Figure 4A). 

Since the entrapped magnetic flux in the superconducting magnet is one of the common extrinsic factors for the distortion of the AC field waveform, thus leading to even harmonic generation, we have performed an additional experiment without the zero-field shield option. Expectedly, as shown in the inset of Figure 4A, under identical conditions ($H_{ac}=1\;Oe\;(80\;A/m)$ and f=333 Hz), the $\left|\chi_{2}\right|$ signal reappears with a much higher intensity. 

Taking into consideration the dual superconducting and ferromagnetic properties of the FeSe system, we can presume that the resulting internal static and dynamic field profiles in the crystal are quite complicated and also can lead to even harmonic development. Based on our previous DC magnetometric analysis of the studied samples, we have to note the typically hard magnetic hysteresis with high remanence and coercivity values, together with the observed thermomagnetic irreversibility in the ZFC–FC measurements at the temperature interval 2–12 K [37,56]. 

Moreover, the magnetic properties of FeSe have been thoroughly analyzed in the literature, both in the context of its intrinsic nature with vacancy induced magnetic clusters [57] and sophisticated phase diagrams with nematic, stripe-order states and prominent low-energy Neel-type spin fluctuations [58]. The influence of foreign ferro- and ferrimagnetic phases (hexagonal Fe_7_Se_8_ in our case) has been extensively studied as well. In addition to the sample’s complex crystallographic structure [42], the off-axis applied AC field orientation ($H_{ac}⊥[101]$ FeSe crystal plane) affects the asymmetric intrinsic field profile. This was recently discussed by Ivan et al. [59] as another possible source for the second harmonic generation. The behavior of the second harmonic at different frequencies is also presented in Figure 4B.

With the application of higher frequencies, an increase in the $\left|\chi_{2}\right|$ modulus peak is detected. In general, the effects of frequency variation over even harmonics are still undetermined, and to our knowledge, there are just a few studies in the literature.

In this context, the detected frequency tendencies of $\left|\chi_{2}\right|$ for the investigated FeSe crystal are analogous to the available information for the cuprates [51]. This is a generally unexplored research direction with presently limited options for more conclusive experimental and theoretical model comparisons, and further detailed studies are required. 

The variable behavior of the even (second) harmonic (depending on the $H_{ac}/H_{dc}$ ratio) has already applied in some pragmatic options for superconductor characterization and practical applications as well. The sensitive field dependencies of $\chi_{2}$ effectively can trace down the gradual thermomagnetic evolution in the initial magnetic flux penetration in the transition from a diamagnetic Meissner (reversible) state to the first vortex entering and pinning (irreversible). However, for a superconductor with a particular granular morphology [52] and/or internal-void structure, the field penetration process can be much more sophisticated. A prominent example is the establishment of a paramagnetic Meissner (Wohlleben) effect [60]. It is based on the magnetic reentrance in the superconductor transition and formed due to interactions between the trapped remanence field profile in the sample, bulk superconductivity effects and especially the complex weak-link networktypes of Josephson junctions (π−π) and the symmetry of the superconducting order parameter (*s*-wave, *d*-wave). Therefore, these processes are extensively investigated by means of complex AC magnetic susceptibility [53,54]. The second harmonic is used for a precise $H_{c1}$ estimation [61] and to examine the changes and memory effects in the internal remanence field profile and Josephson network nonlinear behavior [62]. An additional example for the practical development of $\chi_{2}$ sensitivity based on the $H_{ac}/H_{dc}$ ratio and marked by the maximal harmonic signal is demonstrated for sensor applications with the development of superconducting fluxgate magnetometers [33]. In this context, the even harmonic response of the FeSe system and generally of iron-based superconductors (with a prominent magnetic nature) can be also utilized in such practical directions. 

The third harmonic behavior has been extensively analyzed both experimentally and theoretically upon the fundamental concepts of the critical-state models, the vortex diffusion dynamics under the frames of the dissipative process and the phase transitions in the vortex matter. Its generally oscillating behavior reflects the balance between the vortex-matter stability through pinning against the dynamics and corresponding losses in the superconductor at given experimental conditions [12]. This is well presented for the FeSe crystals with the temperature behavior of the in-phase and out-phase components, as shown on Figure 5.

The positive values of ${\chi \prime }_{3}\left(T\right)$ are an accurate indicator for the establishment of the critical state, and the negative values mark the dissipative effects. Usually, the ${\chi^{\prime \prime }}_{3}(T)$ shape also follows a mirror change with the same phenomenology. 

**Figure 6:**
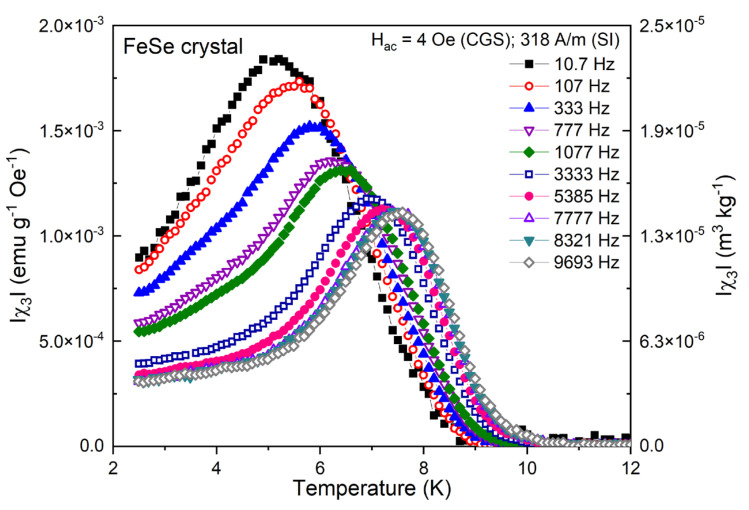
Temperature dependence of $\left|\chi_{3}\right|$ at fixed $H_{ac}=4\;Oe\;(318\;A/m)$ and different frequencies for the FeSe crystal.

For a more precise quantitative analysis of the pinning characteristics and the critical current, we have used the parametric dependencies on the $\left|\chi_{3}\right|$ modulus peak. The flux creep is the main dynamic source contributing to the nonlinear/hysteretic behavior and the frequency-variation effect over the $\left|\chi_{3}\right|$ peak, as the point of full field penetration for reaching the critical current density $J≅J_{c}$ provides an additional accurate criterion for the estimation of the pinning activation energy and its functional dependencies. The measured $\left|\chi_{3}\right|$ harmonic susceptibility curves as a function of temperature are presented in Figure 6 for different frequencies. It is important to note that a frequency increase results in a contrastive $\left|\chi_{3}\right|$ peak tendency compared to ${\chi \prime \prime }_{1}$, in terms of decreasing intensity as a consequence of the gradual transitions through the critical state (stable pinning) at the lowest frequencies, the flux creep in the intermediate regime and the dominant flux flow at the highest frequencies. The frequency dependence of third harmonic susceptibility is very strong (the observed temperature shift of the $\left|\chi_{3}\right|$ peak in the analyzed frequency range is $\Delta T_{p}$ shift ~ 2.5 K for $H_{ac}=4\;Oe$ (318 A/m)) across the entire temperature interval, thus marking a significant dynamic process influence also as a consequence of the rapid decrease in the pinning energy $U_{p}$ when the temperature approaches $T_{c}$, due to a reduced vortex pinning strength in the superconducting fraction of the crystal.

**Figure 7:**
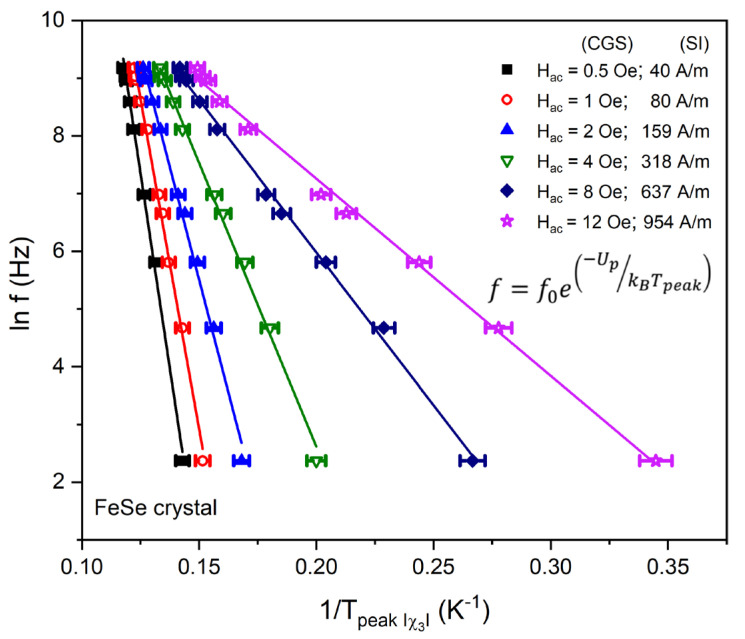
Arrhenius plots: logarithmic-scale representation of frequency (ln *f*) as a function of the inverse of the peak temperature 1/*T_peak_* of $\left|\chi_{3}\right|$ at different AC fields through linear data fits.

The acquired data for the frequency variation of the peak temperature $T_{p}$ of $\left|\chi_{3}\right|$ (at different AC field amplitudes) are plotted following the semi-log Kim–Anderson Arrhenius-type frequency relationship:

(1) f=f0e−UpkBTpeak

where $U_{p}$ is the pinning energy, $f_{0}$ is a characteristic attempt frequency (typically in the range $10^{9}–10^{12}\;Hz$) and $\kappa_{B}$ is the Boltzmann constant. $U_{p}$ is estimated from the slope in the linear fit, as shown in Figure 7. The $\left|\chi_{3}\right|$ modulus peak also marks the point of the complete AC field penetration, where the flux front reaches the sample center and the maximal critical current [63,64,65]. This allows also the determination of the activation energy as a function of the applied $H_{ac}$ field and the construction of the equivalent $U_{p}\sim J_{c}^{-\mu }$ dependencies, where the critical “glassy” exponent (μ=17; 52 and 79) describes the corresponding single vortex, small or large vortex, bundle within the frames of collective pinning theory [66,67]. Taking into account the magnetic field orientation perpendicular to the crystal plane (thin plate-like geometry) and the deformed self-field profile, the critical current density $J_{c}$ can be extracted using Expression (2) [68] related to the applied AC field amplitude $H_{ac}$ and the crystal dimensions a and d.

(2) $$H_{AC}^{Peak}=J_{C}\frac{d}{\pi }\left[\frac{2a}{d}\arctan\frac{d}{a}+\ln\left(1+{\left(\frac{a}{d}\right)}^{2}\right)\right]$$

**Figure 8:**
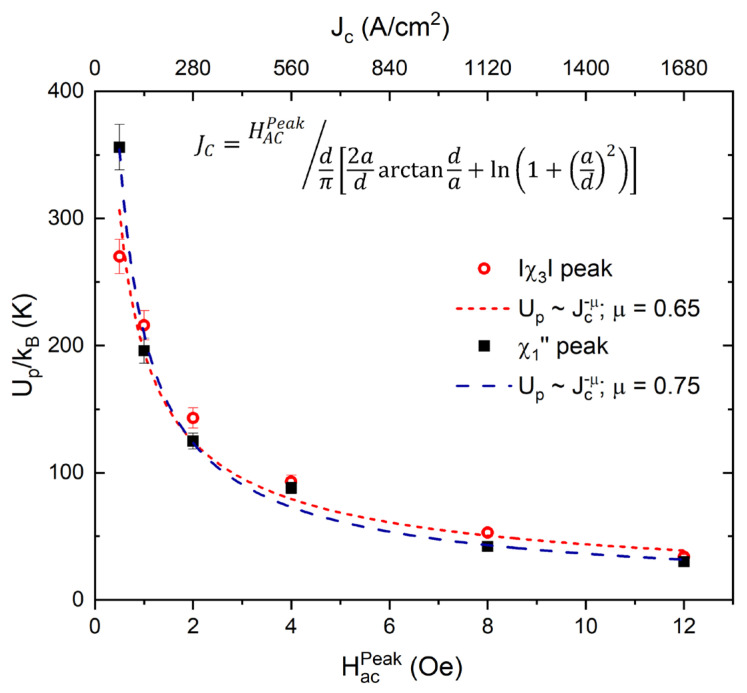
AC field/critical current dependence of the thermal activation energy $U_{p}$ determined for both ${\chi \prime \prime }_{1}$ and $\left|\chi_{3}\right|$. The dashed lines are the power law fit of the experimental data corresponding to the collective creep of large vortex bundles.

A well-scaled power law approximation of the $U_{p}\left(J\right)$ data was identified and is presented in Figure 8 for both ${\chi \prime \prime }_{1}$ and $\left|\chi_{3}\right|$. 

With a simple data-fit routine, we estimated the critical exponent μ=0.6−0.7≈7/9, which is typical for unconventional superconductors, where intense thermal fluctuations and reduced $U_{p}$ potential results in a collective creep of large-size vortex bundles [66,67].

## 4. Conclusions 

In concluding remarks, we have studied the nonlinear AC magnetic susceptibility of flux-grown FeSe crystals. For that purpose, the AC magnetic response was explored with various experimental series in a broad range of AC field amplitudes and frequencies. Through the development of a combined analysis of fundamental and higher harmonic components, we have observed several unexplored features in the AC magnetic response:

1. The unusual detection of second order harmonic components, even without a superimposed DC field. We have discussed that the origin of $\chi_{2}$ generation, as a mark of asymmetricity in the AC magnetization cycle, is possibly related to the presence of both dynamic and static magnetization intrinsic profiles due to the dual superconducting/magnetic nature and structural complexity of the crystal;
2. The comparative analysis of the fundamental and third harmonic components revealed a temperature divergence in the activity of the linear/nonlinear vortex dynamics (without a superimposed DC field). Similarly, as for the second harmonic, this also can be related to the indirect influence of the intrinsic magnetic order.

In addition, the core phenomenological concepts of the odd- and even harmonic generation were discussed in the context of the vortex-matter state, dynamics and properties. The basics of the pinning energy estimation using the Kim–Anderson Arrhenius relation of the ACMS frequency dependencies were revisited. 

Following a simplified approach, we have innovatively utilized the ACMS third-harmonic analysis as a sensitive marker of the nonlinear dynamical process activity for effective and accurate $U_{p}$ evaluation. The functional dependencies of the pinning barrier were determined revealing a power law $U_{p}\sim J_{c}^{-\mu }$ relation within the frames of a strong thermally activated collective creep (large bundles) typical for nonconventional superconducting systems with a reduced pinning strength, as in the case of the studied FeSe crystals. 

To our knowledge, the observed higher harmonic features are unexplored and highlight novel prospects for the further development of the ACMS technique. The presented combined ACMS fundamental and higher harmonic ($\chi_{2}$ and $\chi_{3}$) analysis demonstrates the versatility of the technique and its ability to provide qualitative and quantitative information on the basic mixed state parameters and a universal approach for the characterization of superconducting materials.

## Data Availability

The datasets that support the findings in this study are available from the corresponding authors upon reasonable request.
